# Supplementary material for: Influenza A Virus Infection Reactivates Human Endogenous Retroviruses Associated with Modulation of Antiviral Immunity
Source: Viruses. 2022 Jul 21;14(7):1591. doi: 10.3390/v14071591 (PMC9320126; doi:10.3390/v14071591)
Supplement: Supplementary file 1 [file viruses-14-01591-s001.zip › MotifAnalysis.zip!/MotifOutput_SC35M/homerResults/motif6.similar.html]

motif6

## Information for motif6

C
T
G
A
A
C
T
G
A
C
T
G
C
G
A
T
A
C
G
T
A
C
G
T
A
C
T
G
A
C
G
T
C
T
G
A
C
G
T
A
  
Reverse Opposite:  

A
C
G
T
A
G
C
T
C
G
T
A
A
G
T
C
C
G
T
A
C
G
T
A
C
G
T
A
A
G
T
C
A
G
T
C
A
G
C
T
  

|  |  |
| --- | --- |
| p-value: | 1e-15 |
| log p-value: | -3.642e+01 |
| Information Content per bp: | 1.906 |
| Number of Target Sequences with motif | 9.0 |
| Percentage of Target Sequences with motif | 21.95% |
| Number of Background Sequences with motif | 93.2 |
| Percentage of Background Sequences with motif | 0.20% |
| Average Position of motif in Targets | 120.8 +/- 47.5bp |
| Average Position of motif in Background | 98.5 +/- 87.6bp |
| Strand Bias (log2 ratio + to - strand density) | 10.0 |
| Multiplicity (# of sites on avg that occur together) | 1.00 |
| Motif File: | file (matrix) reverse opposite |

### Similar de novo motifs found

|  |  |  |  |  |  |  |  |
| --- | --- | --- | --- | --- | --- | --- | --- |
| Rank | Match Score | Redundant Motif | P-value | log P-value | % of Targets | % of Background | Motif file |
| 1 | 0.843 | A T C G A C T G C G T A A C G T A C G T A C T G A C G T C G T A | 1e-15 | -35.393219 | 31.71% | 1.10% | motif file (matrix) |
| 2 | 0.618 | G C T A C G T A T G C A T A C G A T C G T C A G C G T A C G A T C G A T T A C G C A G T G T A C | 1e-15 | -35.102419 | 24.39% | 0.38% | motif file (matrix) |
| 3 | 0.605 | A G C T A C G T A T C G A C G T C T G A C G T A C T G A A G T C C T A G A G T C | 1e-10 | -25.065940 | 21.95% | 0.71% | motif file (matrix) |
| 4 | 0.804 | A C T G A C G T A C G T A C G T A C T G A C G T A C T G C G T A | 1e-5 | -12.189837 | 12.20% | 0.61% | motif file (matrix) |
